# Virtual Walking Sensation by Prerecorded Oscillating Optic Flow and Synchronous Foot Vibration

**DOI:** 10.1177/2041669519882448

**Published:** 2019-10-15

**Authors:** Michiteru Kitazaki, Takeo Hamada, Katsuya Yoshiho, Ryota Kondo, Tomohiro Amemiya, Koichi Hirota, Yasushi Ikei

**Affiliations:** Department of Computer Science and Engineering, Toyohashi University of Technology, Japan; Interfaculty Initiative in Information Studies, The University of Tokyo, Japan; Department of Computer Science and Engineering, Toyohashi University of Technology, Japan; The Graduate School of Information Science and Technology, The University of Tokyo, Japan; The University of Electro-Communications, Tokyo, Japan; Tokyo Metropolitan University, Japan

**Keywords:** optic flow, self-motion, vection, walking, jitter, tactile vibration

## Abstract

This article reports the first psychological evidence that the combination of oscillating optic flow and synchronous foot vibration evokes a walking sensation. In this study, we first captured a walker’s first-person-view scenes with footstep timings. Participants observed the naturally oscillating scenes on a head-mounted display with vibrations on their feet and rated walking-related sensations using a Visual Analogue Scale. They perceived stronger sensations of self-motion, walking, leg action, and telepresence from the oscillating visual flow with foot vibrations than with randomized-timing vibrations or without vibrations. The artificial delay of foot vibrations with respect to the scenes diminished the walking-related sensations. These results suggest that the oscillating visual scenes and synchronous foot vibrations are effective for creating virtual walking sensations.

## Introduction

Walking is a natural and frequent action performed by healthy adults in everyday life. It involves various sensations as well as motor commands and actions. During walking, a person moves their legs and arms and strikes the ground with their feet. At the same time, they perceive vestibular sensations and proprioception, observe visual motion flow, hear changing sounds, feel airflows on the skin, experience smell, and receive tactile sensations on the feet. We are motivated to develop a virtual reality (VR) system that can present experiences of walking to persons who are at a distance or have a disability that prevents them from walking. The virtual walking system would enable people to walk on strange places such as the moon or the ocean floor and improve the quality of life of people who have walking disabilities in future. As the first step for it, we aim to create a virtual sensation of walking using limited modalities such as vision and tactile sensations.

Visual motion flow or optic flow is one of the most extensively studied stimuli for investigating self-motion. Optic flow contains information of self-motion as well as object and environment motions and structures (Banton, Stefanucci, Durgin, Fass, & Proffitt, 2005; Gibson, 1966, 1968; Kitazaki & Shimojo, 1998; Nakayama, 1985). Vection can be defined as a visually induced illusory self-motion perception. It is an important component of the walking sensation. The definition of vection has been comprehensively discussed and updated by Palmisano, Allison, Schira, and Barry (2015). Definitions of vection are categorized into four groups: (a) visual illusion of self-motion in a stationary observer, (b) modality-independent illusion of self-motion, (c) visually mediated perception of self-motion in either reality or illusion, and (d) real or illusory conscious subjective experience of self-motion. The first one is the narrowest category, while the last one is the broadest. The feeling of self-motion during real walking is included in the fourth definition. In this study, we strived to utilize vection in terms of the first definition (visually induced self-motion illusion) and the second definition (self-motion illusion from vision and tactile sensation on feet) to make a virtual walking system for stationary observers.

Vection is dominated by background motion (Brandt, Wist, & Dichgans, 1975; Ohmi, Howard, & Landolt, 1987) and nonattended motions (Kitazaki & Sato, 2003), and it is enhanced by enlarging the field of view (Dichgans & Brandt, 1978), binocular stereopsis (Allison, Ash, & Palmisano, 2014; Palmisano, 1996, 2002), and adding perspective jitter on the radial optic flow (Palmisano, Allison, Ash, Nakamura, & Apthorp, 2014; Palmisano, Allison, Kim, & Bonato, 2011; Palmisano, Burke, & Allison, 2003; Palmisano, Gillam, & Blackburn, 2000). This perspective jitter is similar to the oscillation of the visual scene during actual walking. The sensations of walking and vection are improved by adding oscillating patterns of optic flow based on motions of the walker’s head and eye motions (Bubka & Bonato, 2010; Lécuyer, Burkhardt, Henaff, & Donikian, 2006). However, the added jitter is not required to be realistic for enhancing vection (Palmisano et al., 2014). Vection is inhibited during walking on a treadmill (Ash, Palmisano, Apthorp, & Allison, 2013; Onimaru, Sato, & Kitazaki, 2010). Contrary to these studies, a study reported that forward vection was enhanced by forward walking (Seno, Ito, & Sunaga, 2011). In the study, the speed of optic flow (57.6 km/hour) and the speed of treadmill (2 km/hour) were very different, although the speed of optic flow was matched or similar to the speed of treadmill in the other studies. A simulated viewpoint jitter enhances vection even during walking (Ash et al., 2013). Thus, we predicted that the oscillation of a visual scene simulating the eye and head motion would contribute to the sensation of walking.

In VR research, various systems have been developed for presenting the sensation of walking. Omnidirectional treadmills enable users to walk in any direction in one place (Iwata, 1999), while leg-support actuator systems enable users to walk and navigate up or down stairs (Iwata, Yano, & Nakaizumi, 2001). These VR systems focus on leg movements and the motor commands required to walk in the real world. By combining the systems with a display, such as a head-mounted display (HMD) or a large projection screen, walking experiences have been created in VR studies. Based on the first vection study using a new-generation HMD that has a large visual field (>90°), very fast sampling of head motion (1 kHz), and immediately synchronized visual updating, it was reported that visual compensation with head motion improved the vection sensation (Kim, Chung, Nakamura, Palmisano, & Khuu, 2015).

Rhythmic stimulation to the feet may induce spinal central pattern generators to produce an active walking sensation, which is expected to contribute to walking rehabilitation (Chéron et al., 2012; Gravano et al., 2011). A VR system was developed by utilizing rhythmic stimulations on the feet and small movements of the feet, legs, and trunk enforced by actuators with multisensory presentations of airflow, smell, changing sounds, and three-dimensional video images (Ikei, Abe, Hirota, & Amemiya, 2012; Ikei et al., 2015). However, there is no psychological evidence on the strength of sensory perception of walking by users and the critical factors affecting the walking sensations.

In this study, we strived to identify the critical parameters for enabling stationary observers to experience virtual walking without leg action. We focused on tactile sensations on the feet and oscillating or jittering optic flow. We developed a VR system with a large-field-of-view HMD and the ability to produce four-channel vibrations on the forefeet and heels of both feet. In the experiments, we captured actual walking scenes with footstep timings and measured the psychological responses to walking-related sensations. The visual oscillation caused by the walker’s actual head motion was included in the stimuli, and the image had a binocular disparity. However, the visual compensation of the observer’s head motion was not implemented so that they could not gaze around the scene.

## Experiment 1

### Methods

#### Participants

Fifteen undergraduate and graduate students (all males, mean age of 21.35 years, ±0.88 standard deviation) participated in Experiment 1. All participants provided written informed consent and had normal or corrected-to-normal vision. The methods of the experiment and all experimental protocols were approved by the Ethical Committee for Human-Subject Research at the Toyohashi University of Technology. The experiments were strictly conducted in accordance with the approved guidelines of the committee and the Code of Ethics of the World Medical Association (Declaration of Helsinki).

#### Stimuli and apparatus

**Figure 1.**
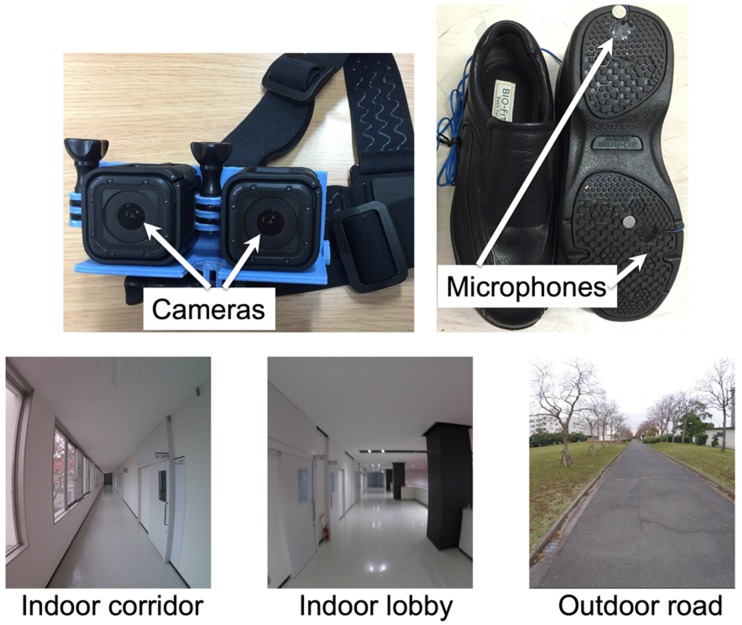
Stereo camera device for capturing stereo motion images (top-left). A pair of shoes with microphones for capturing timings of footsteps (top-right). Three locations where the walking scenes were captured (bottom).

Two cameras (GoPro HERO 4 Session, 2,560 [height] × 1,440 [width] pixels, 122.6° × 94.4°, 30 fps, 65-mm intercamera distance (Dodgson, 2004); Figure 1, top-left) were mounted on the forehead to capture binocular-stereo first-person-view optic flow. Four small condenser microphones (SP Limited, XCM6035) were embedded in the soles of a pair of shoes to obtain footstep timings (left and right heels and forefeet; Figure 3, top-right). Two walkers wore these cameras (camera viewpoint height: 169.1 and 172.0 cm) and shoes and walked at three different locations (a corridor in a school building, a lobby in a school building, and an outdoor paved road in the university campus; Figure 3, bottom). These locations were familiar to the participants. To exclude the possibility of artifacts caused by a specific scene or situation, we used three different scenes. To exclude the possibility of artifacts caused by a specific walker or walking movement, we used two different walkers. Walkers stomped at one place for four steps while observing their feet at the beginning, after which they gazed forward and walked straight.

**Figure 2.**
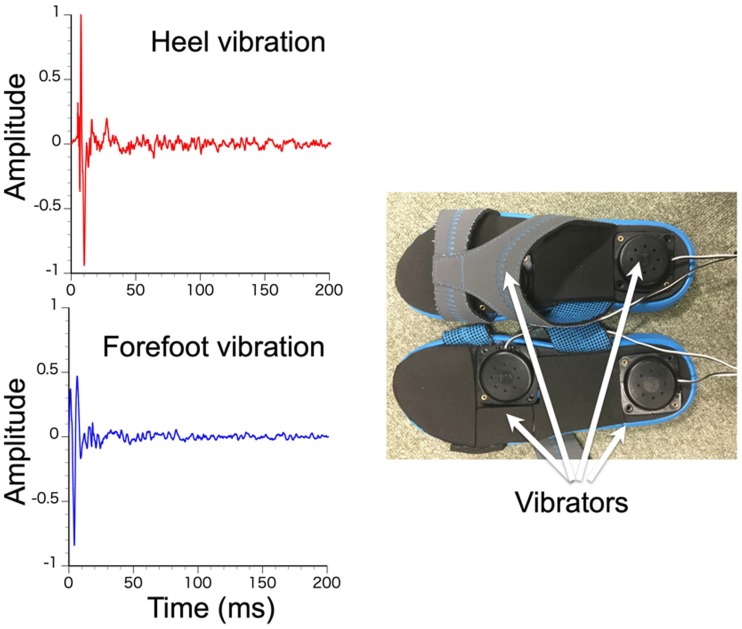
Profiles of presented vibrations to heel and forefoot (left). Experimental apparatus for tactile stimuli (right).

The timings of heels and forefeet strikes on the ground were extracted from the sounds by applying a high-pass filter at 2.1 kHz and visual and hearing inspections. Walkers were asked to walk at 2 steps/second after training. Foot vibrations were produced by applying a low-pass filter at 240 Hz to the sounds of real footsteps on a paved road surface. Vibrations were 200-ms long and different for the forefoot and heel (Figure 2, left). Stereo motion images were presented on an HMD (Oculus Rift DK2, 960 [width] × 1,080 [height] pixels, 90° × 110°, refresh rate of 60 Hz). Captured images were appropriately trimmed and formatted for the HMD to exclude visual discrepancies. Vibrations (200-ms duration) were presented on the heels and forefeet of the observer at the actual timings of foot strikes (Vibrotactile device Acouve Lab Vp408; Figure 2, right). A computer (Intel Core i7-4790 CPU @ 3.60 GHz, NVidia GeForce GTX 745) controlled the visual stimuli on the HMD and the tactile stimuli on the vibrotactile devices. Vibrations were presented on the vibrotactile devices by inputting sound signals from a power amplifier (Behringer EPQ450, 4 0W (8Ω) × 4 ch) through a USB multichannel preamplifier (Behringer FCA1616, input 16 ch, output 16 ch) controlled by the computer. The vibrations to the heel and the forefoot were presented at the timing extracted from the actual walking.

#### Design

Experiment 1 contained three repetitions of all combinations of three vibration conditions (synchronous, random, and no vibration), two walkers, and three locations (54 trials in total). The frequency of random vibrations was identical to that of synchronous vibrations; however, its presentation timings were randomized.

#### Procedure

Participants observed each stimulus for 20 seconds, after which they were asked to rate the sensation strengths of (a) self-motion (vection), (b) walking, (c) leg action (footstep), and (d) telepresence by using Visual Analogue Scale (VAS). We explained these sensations to participants as follows. If the participants feel as if they were passively moving, it is a self-motion sensation. If the participants feel as if they were walking, it is a walking sensation. If the participants feel as if they were stamping or stepping on the ground, it is a leg-action sensation. If the participants feel as if they were physically present in the visual scenes, it is telepresence. Although presence has different definitions (Skarbez, Brooks, & Whitton, 2017), in this study, we use the term *telepresence* in the sense of spatial presence at the place in video images. They were seated during all the experiments and asked not to move their body or head for all the trials. In all the experiments, before the actual trials, they experienced several trials as a practice session in which a different scene was used.

**Figure 3.**
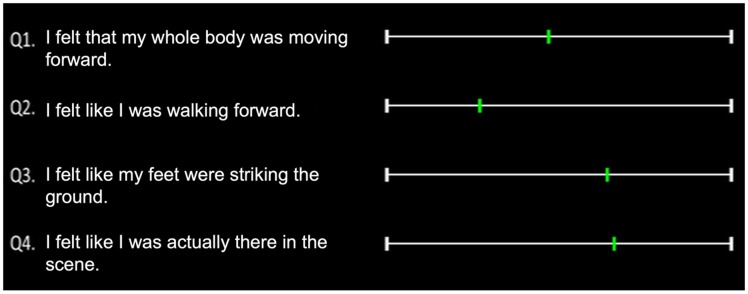
An example of a screen for VAS ratings.

Four sentences regarding these sensations were presented on the screen after each stimulus presentation: Question 1: I felt that my whole body was moving forward; Question 2: I felt like I was walking forward; Question 3: I felt like my feet were striking the ground; and Question 4: I felt like I was actually there in the scene. The order of questions was constant though all trials in the experiment. Lines and cursors for the VAS ratings were placed on the right side of each question (Figure 3). The data were converted into a numerical scale ranging from 0 to 100. Participants were informed that the left end (0) meant *no sensation* and the right end (100) meant the *same sensation* as in actual walking. They had adequate time to judge all questions without time limitations. During experiments, noise-canceling headphones (Bose Quiet Comfort 2) were used to present white noise (70 dBA) to prevent participants from hearing the vibrotactile devices. The participants’ heads were not strictly fixed. The participants were instructed not to move their heads but to observe the stimuli from a relaxed state.

### Results

After observing dynamic scenes while receiving vibrations on the feet, participants rated the strength of perceived self-motion, walking sensation, leg-action sensation, and telepresence using a VAS. Self-motion refers to the sense of passive motion similar to vection. If the participants felt as if they were walking, they experienced a walking sensation. If the participants felt as if they were stamping or stepping on the ground, they experienced a leg-action sensation. If the participants felt as if they were physically present in the visual scenes, they experienced telepresence.

**Figure 4.**
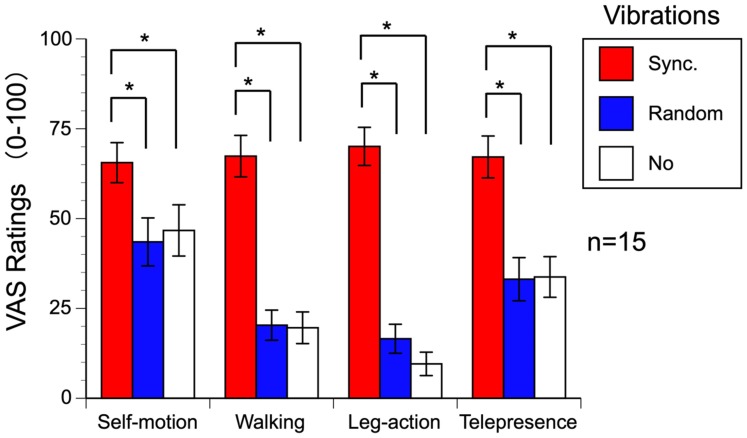
Results of Experiment 1. Averaged VAS ratings among participants are plotted, and vertical error bars indicate the standard error of the mean. VAS = Visual Analogue Scale.

Self-motion, walking sensation, leg-action sensation, and telepresence were all rated significantly higher by observing a walker’s first-person-view scenes that included the actual oscillation or jittering of the walker’s head position with synchronized vibrations on the feet (heels and forefeet) than with randomized-timing vibrations or without vibrations (Figure 4). We conducted three-way repeated-measure analyses of variance with vibration conditions (synchronous, random, and no vibrations), scenes (three different locations), and walkers (two persons with 169.1 cm and 172.0 cm heights) as the factors using digitized VAS data (0–100; 0 = *no sense*, 100 = *identical to actual walking in the real world*) for self-motion, walking sensation, leg-action sensation, and telepresence.

All the main effects of the vibration conditions showed statistical significance, self-motion: *F*(2, 28) = 16.701, *p* < .001, ${\text{η}}_{\text{p}}^{\text{2}}$ = .544; walking sensation: *F*(2, 28) = 51.771, *p* < .001, ${\text{η}}_{\text{p}}^{\text{2}}$ = .787; leg-action sensation: *F*(2, 28) = 80.906, *p* < .001, ${\text{η}}_{\text{p}}^{\text{2}}$ = .852; and telepresence: *F*(2, 28) = 20.523, *p* <.001, ${\text{η}}_{\text{p}}^{\text{2}}$ = .594. Multiple comparisons showed that the synchronized foot vibrations elicited stronger sensations of self-motion, walking, leg action, and telepresence than the randomized-vibration or no-vibration conditions (Shaffer’s *F*-modified sequentially rejective Bonferroni procedure *p*s < .05). There was no difference between the randomized-vibration and no-vibration conditions. Thus, the combination of oscillating optic flow with synchronized vibrations or vibrations at the actual timings on the feet was necessary to enhance the virtual walking experience.

The main effect of the walker conditions was significant for the leg-action sensation, *F*(1, 14) = 6.762, *p* = .021, ${\text{η}}_{\text{p}}^{\text{2}}$ = .326, and the rating was higher with the shorter walker’s stimuli than the taller walker’s stimuli. We obtained no other main effects or interactions.

We found that the oscillating optic flow with synchronized vibrations on the feet was critical to enhance the virtual walking experience in comparison with the random vibrations or without vibrations. However, it is not clear how much we are sensitive to synchronization of visual oscillation and vibrations for the virtual walking. Thus, in the next experiment, we investigated the effect of phase delay of the vibrations.

## Experiment 2

### Methods

#### Participants

Fifteen undergraduate and graduate students (1 female and 14 males, mean age of 20.7 years, ±1.3 standard deviation) participated in Experiment 2. None of them participated in Experiment 1. All participants provided written informed consent and had normal or corrected-to-normal vision. The methods of the experiment and all experimental protocols were approved by the Ethical Committee for Human-Subject Research at the Toyohashi University of Technology. The experiments were strictly conducted in accordance with the approved guidelines of the committee.

#### Stimuli and apparatus

The stimuli and apparatus were identical to Experiment1.

#### Design

Experiment 2 contained three repetitions of all combinations of three vibration-delay conditions (0, 0.25, and 0.5 phase-delayed vibrations), two walkers, and three locations (54 trials in total). The conditions of 0.25 and 0.5 phase delay approximately correspond to 250- and 500-millisecond delay sounds, respectively. The 0.5 phase delay implied that the left and right feet vibrations were almost reversed with respect to the scene oscillation because the walkers in the scene walked at 2 steps/second.

#### Procedure

Participants performed the same task as in Experiment 1; they observed each stimulus for 20 seconds, after which they were asked to rate the sensation strengths of (a) self-motion (vection), (b) walking, (c) leg action (footstep), and (d) telepresence by using VAS.

### Results

**Figure 5.**
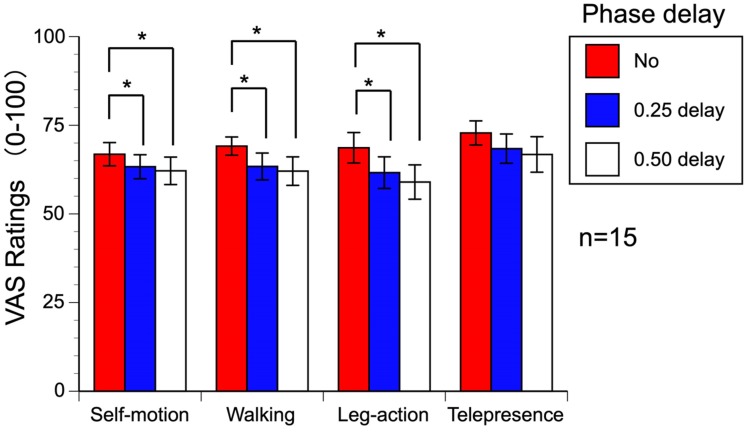
Results of Experiment 2. VAS = Visual Analogue Scale.

Self-motion, walking sensation, and leg-action sensation significantly decreased with 0.25 and 0.5 phase-delayed (250 and 500 milliseconds) vibrations on the feet in a comparison with the synchronized (no delay) condition when observing the walker’s oscillating first-person-view scenes (Figure 5). We conducted three-way repeated-measure analyses of variance with the vibration-delay conditions (synchronous, 0.25 phase delay, and 0.5 phase delay), scenes (three different locations), and walkers (two persons with 169.1 cm and 172.0 cm heights) as the factors using digitized VAS data for all sensations.

Statistical significance of the main effect of the vibration-delay condition was obtained for the sensation of self-motion, *F*(2, 28) = 4.679, *p* = .018, ${\text{η}}_{\text{p}}^{\text{2}}$ = .251, walking, *F*(2, 28) = 5.402, *p* = .010, ${\text{η}}_{\text{p}}^{\text{2}}$ = .278, and leg action, *F*(2, 28) = 4.656, *p* = .018, ${\text{η}}_{\text{p}}^{\text{2}}$ = .250; however, no statistical significance was obtained for telepresence, *F*(2, 28) = 3.098, *p* = .061, ${\text{η}}_{\text{p}}^{\text{2}}$ = .181. Multiple comparisons showed that the synchronized foot vibrations elicited stronger sensations of self-motion, walking, and leg action than the 0.25 or 0.5 phase-delay conditions (*p*s < .05). There was no difference between the 0.25 and 0.5 phase-delay conditions. Thus, the consistency of timings of foot vibrations and scene oscillations affected the strength of walking-related sensations. However, the effect of phase delay was not very significant.

The main effect of the scene conditions was significant for the leg-action sensation, *F*(1, 14) = 4.330, *p* = .023, ${\text{η}}_{\text{p}}^{\text{2}}$ = .236, and the rating was higher with the outdoor road scene than the indoor corridor and lobby scenes. However, multiple comparisons showed no significant differences between the scenes (*p*s > .081). We obtained no other main effects or interactions.

We found that the 0.25 or 0.5 phase delay of foot vibration deteriorated the strength of self-motion, walking and leg-action sensation, but not the telepresence. Thus, not only the rhythmical vibration but also its synchronization to the visual oscillation are necessary for the virtual walking.

## Discussion

### Summary of Results

The captured first-person-view scenes with image oscillations caused by the walker’s head motion and the foot vibrations at synchronized timings induced sensations of self-motion, walking, leg action, and telepresence. The synchronous presentation of visual oscillations and foot vibrations was critical for enhancing the virtual walking experience.

The effect of the foot vibration was notable in all experiments. The foot vibration had to match the actual walking, while the randomized vibrations had no effect. These results suggest that the tactile stimulation on the feet for footsteps is effective for enhancing virtual walking sensations.

### Sensitivity to Phase Delay of Vibrations

The phase delay of foot vibrations to the captured timings of footsteps significantly decreased the sensations of self-motion, walking, and leg action in Experiment 2. The 0.25 and 0.5 phase delays were delays of 250 and 500 milliseconds, respectively. The high sensitivity to such a small discrepancy between visual oscillation and foot tactile sensations may have been related to the reciprocal inhibitory interaction between the visual and vestibular system and the tactile and vestibular system (Berthoz, Pavard, & Young, 1975; Brandt, Bartenstein, Janek, & Dietrich, 1998; Hwang, Agada, Kiemel, & Jeka, 2014; Kleinschimdt et al., 2002; Peterka, 2002; Peterka & Benolken, 1995; Redfern & Furman, 1994; Wong & Frost, 1981). Visual information is dominant in the absence of vestibular information (Berthoz et al., 1975; Brandt et al., 1998; Kleinschimdt et al., 2002; Wong & Frost, 1981), and the visual and somatosensory information is utilized more for postural control in the absence of vestibular information (Dichgans & Brandt, 1978; Hwang et al., 2014; Peterka, 2002; Peterka & Benolken, 1995; Redfern & Furman, 1994). Thus, the sensitivity to vision and touch might be enhanced in the absence of vestibular information in our virtual walking system. Moreover, it is reported that active observers are sensitive to small discrepancies between visual oscillation and their head motion (Ash, Palmisano, Govan, & Kim, 2011). Display lag for active observers who are physically oscillating their head impairs vection if the lag is 50 or 100 milliseconds; however, it does not impair vection if the lag is 200 milliseconds. This finding is consistent with our result. Thus, it is suggested that the strict synchronization of vision and touch contributes to the enhancement of walking-related sensations for stationary observers in the virtual walking system.

### Perceptual Compensation of Visual Oscillation

We seem rarely aware of visual oscillation or image jittering during actual walking because perceptual compensation stabilizes the visual stimuli (Wallach, 1987) and quickly adapts to the environment (Kitazaki, 2013; Wallach & Canal, 1976). Thus, it is suggested that the amplitude of visual oscillation for virtual walking should be as weak as the oscillations perceived during actual walking to obtain the optimal effect.

### Active Walking and Passive Vection

In this study passively seated observers were simulated to be actively walking based on the oscillating optic flow and the foot vibration, and we found that the oscillating optic flow with the foot vibration enhanced the sensation of walking as well as vection. By contrast, in the previous studies (Ash et al., 2013; Onimaru et al., 2010), participants actively walked and passively stood on treadmills, while viewing oscillating and smooth patterns of optic flow, and they have shown that active walking on a treadmill decreases vection (Ash et al., 2013; Onimaru et al., 2010). Thus, one may predict that the foot vibrations enhance illusory perceptions of active walking but interfere with illusory perceptions of self-motion/vection. However, we did not obtain such inhibitory results. Thus, we speculated that the passive sensation of self-motion and the active sensation of walking can be concurrently elicited and can interact with each other in some situations or levels. For example, both occur when we walk on a moving walkway. Vection occurs during walking on a treadmill even though it is weakened by actual walking (Ash et al., 2013; Onimaru et al., 2010). Our virtual walking system probably does not reach the level at which the passive sensation of self-motion is weakened. If the sensation of active walking is significantly increased compared with that in the present system, the sensation of passive self-motion might be decreased. This issue should be further investigated in a future study.

### Dependency on Stimulus Walkers and Scenes

We used two walkers with different heights, and the perception of a three-dimensional scene depends on eye height (Ooi, Wu, & He, 2001; Proffitt, 2006). Actually, we obtained a significant effect of walker height. In Experiment 1, the leg-action sensation was better with the shorter walker’s stimuli than the taller walker’s stimuli. It might have been caused by the difference of heights or the different movements of individuals. Although we did not collect the data of participants’ exact heights, the range was 160 to 180 cm, and the average was approximately 170 cm. In a preliminary experiment using a prototype system (a narrower-FoV HMD with one vibrator each for the left and right heel), we found no correlation of ratings between the walker height and participant height. However, it can be expected that the matching of eye height of the walker and the participant or the normalization of eye height improves the walking sensation. This aspect should be investigated in a future study.

The difference of scenes or locations had some effects on the walking-related sensations, although the effects of visual oscillation and foot vibration were found in all the scenes. In Experiment 2, the leg-action sensation was higher with the outdoor paved road scene than the indoor corridor and lobby scenes (not significant in multiple comparisons). These results suggest that the sensations relating to walking depended on scenes and situations. In this study, we used identical foot-vibration stimuli for all scenes. As the tactile sensation of the feet depends on the type of floor/ground, shoes, walker weight, and other parameters, we should investigate their different effects and optimize the stimuli in a future study. If we could achieve this objective, we would be able to present a variety of experiences of virtual walking to persons who are at a distance or have a disability that prevents them from walking.

### Limitation of Subjective Measurements

One may argue that it is difficult for participants to rate the sensation of walking and leg action using VAS because walking and the leg action are actions and rather implicit in perception. However, as the obtained data were quantitative and consistent among participants, the results of this study seem reliable. In a future, we need to explore behavioral or physiological evidence for virtual walking sensations, rather than subjective evidence using VAS. We have already attempted the proprioceptive self-localization task (Lenggenhager, Tadi, Metzinger, & Blanke, 2007) and jogging after-effect (Anstis, 1995) test after observing the virtual walking stimuli for 60 seconds or 90 seconds. We had expected that the proprioceptive self-location or blind walking at one place would shift in the direction of virtual walking after experiencing the virtual walking with synchronous foot vibrations rather than with randomized vibrations. However, thus far, we found no effect of virtual walking on the self-localization or position shift of blind walking at one place. We plan to measure the cognitive-map performance during virtual walking with and without foot vibrations. We predict that virtual walking may improve the cognitive-map or spatial-memory performance because our spatial representation is effectively updated when we actually move around (Burgess, 2006; Wang & Simons, 1999). 

Furthermore, one may be concerned about the possibility of cross-contamination of four VAS ratings in each trial. We presented all questions on the screen, and we asked participants to rate each one without a time limitation to prevent the cross-contamination of VAS ratings. They were conscious of differences among four ratings because of contrasting questions at a given time. As the resulting ratings were different across conditions, we believe that participants understood the questions appropriately and provided the ratings. However, we could not check all possibilities of cross-contamination in the participants’ subjective judgments. This aspect may have been a limitation of our study. To address this issue, we should try other behavioral measurements to prove the sensation of walking.

## Data Accessibility Statement

The data sets generated and analyzed during this study are available from the corresponding author on a reasonable request.
